# Focal Epithelial Hyperplasia

**DOI:** 10.3390/v13081529

**Published:** 2021-08-02

**Authors:** Simone Kloch Bendtsen, Kathrine Kronberg Jakobsen, Amanda-Louise Fenger Carlander, Christian Grønhøj, Christian von Buchwald

**Affiliations:** Department of Otorhinolaryngology, Head & Neck Surgery & Audiology, Rigshospitalet, Copenhagen University Hospital, Inge Lehmanns Vej 7, 2100 Copenhagen E, Denmark; simone.kloch.bendtsen.01@regionh.dk (S.K.B.); kathrine.kronberg.jakobsen@regionh.dk (K.K.J.); amanda-louise.fenger.carlander@regionh.dk (A.-L.F.C.); christian.groenhoej@regionh.dk (C.G.)

**Keywords:** focal epithelial hyperplasia, FEH, Heck’s disease, multifocal epithelial hyperplasia, MEH, human papillomavirus 13, HPV13, human papillomavirus 32, HPV32

## Abstract

Focal epithelial hyperplasia (FEH) or Heck’s disease is a rare, benign, oral condition that is associated with infection by human papillomavirus type 13, 32 or both. The whiteish to mucosal-colored, soft, papular or nodular elevated lesions in the oral cavity are normally asymptomatic but can grow to a size or at a location where treatment is needed. The diagnosis is often based on clinical presentation and histopathology, and the HPV genotype can be determined using PCR utilizing specific primers or DNA sequencing. While FEH was reported to often affect several members of the same family and exist primarily among indigenous populations around the world, the number of reported cases within the European region is increasing. This contemporary review summarizes the main findings in relation to HPV genotypes, impact of superinfection exclusion and vaccination, transmission, diagnosis, geographical and ethnical distribution, comorbidities and treatment of FEH with an emphasis on including the most recent case reports within the field. Furthermore, we describe for the first time a FEH lesion infected with the low-risk HPV90.

## 1. Introduction

**Figure 1:**
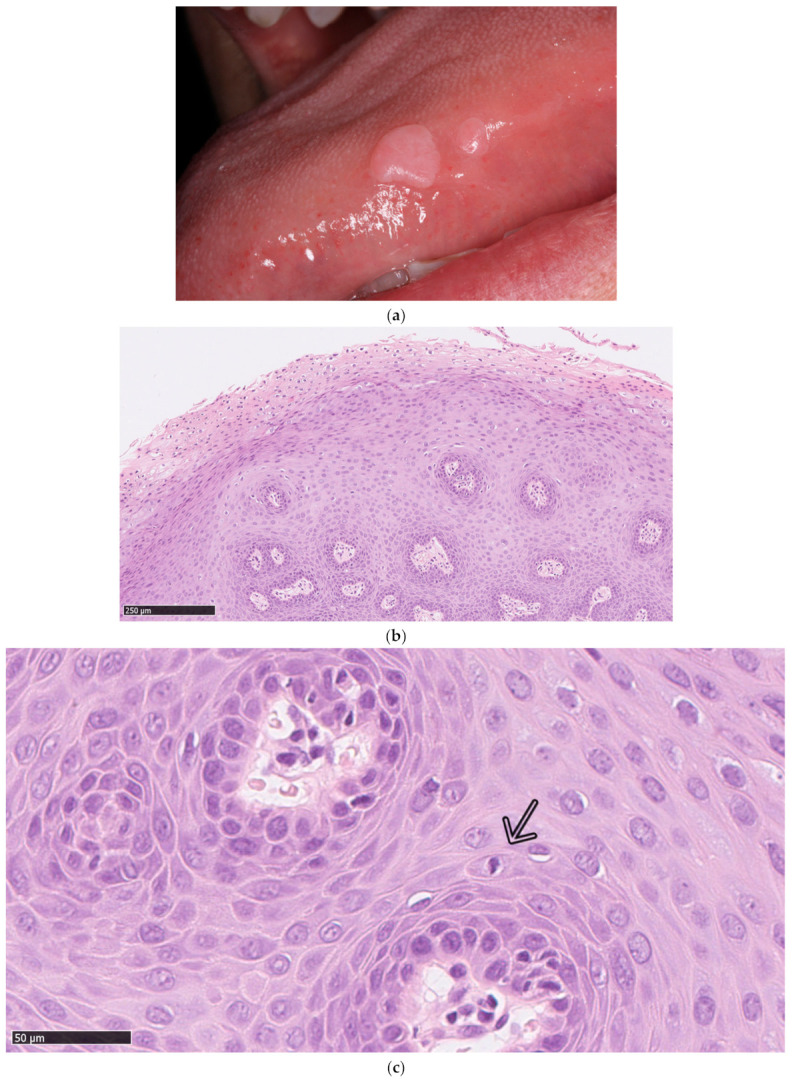
Clinical manifestations of focal epithelial hyperplasia (FEH). (**a**) Clinical photo of two minor FEH lesions located on the lateral side of the tongue. (**b**) Example of a histopathological examination of an FEH lesion (H&E stain) from a 56-year-old Danish female. The lesion was located on the inside of the lower lip and the diagnosis was based on the clinical and histopathological examination. The biopsy revealed a small, flat squamous papilloma with conspicuous koilocytosis that was characterized by several layers of cells with hyperchromatic nuclei with irregular contours and a perinuclear halo. HPV90 was detected with PCR using the VisionArray HPV Chip 1.0. There were no signs of dysplasia or malignancy. (**c**) Example of a mitosoid body (indicated by an arrow) from the FEH lesion described in (**b**).

Focal epithelial hyperplasia (FEH), also known as Heck’s disease or multifocal epithelial hyperplasia (MEH) is a rare, benign oral condition that is induced by infection with human papillomavirus type 13 (HPV13), 32 (HPV32) or both [1]. The condition clinically presents as multiple 0.2–3 cm [2], whiteish to mucosal-colored, soft papular or nodular, elevated lesions in the oral cavity [3,4,5,6] (Figure 1a), which disappear when the mucosa is stretched [7]. While many cases have been reported to be asymptomatic [2,5,6,8,9,10,11,12,13,14], some lesions grow to a size or at a location where treatment is recommended [5,8,9,15,16,17]. A recent systematic review assessed the age of published cases from 1966–2020, and found a wide range of 3–92 years, with a higher reported rate in younger individuals (mean 23.1 years) and a male-to-female ratio of 3:4 [1].

## 2. Human Papillomavirus Genotypes in Focal Epithelial Hyperplasia

HPV13 and HPV32 are low-risk, non-oncogenic and the most prevalent HPV types found in FEH [1,18,19]. However, co-infection or infection with other HPV-types like HPV6, 11, 16, 18, 31, 39, 40, 51, 52, 55, 58, 66, 68, 69, 71 and 74 were also described [4,18,20,21]. Figure 1b,c shows the histology of a FEH lesion from a 56-year-old Danish female infected with the low-risk HPV90. To our knowledge, this is the first report of FEH involving this genotype (Figure 1b,c).

Although multiple FEH cases with high-risk HPV coinfections were reported, to this end, no case of malignant transformation in FEH has been described. Furthermore, an in vitro study confirmed that HPV32 did not have tumor-transforming properties [22]. This is despite the fact that HPV32 is genetically more closely related to the high-risk HPV16 than to the low-risk HPV13 [23]. In the Finnish Family HPV study, the investigators found that a persistent, asymptomatic oral HPV16 infection was associated with HPV16 genomic integration, which might predispose the infected individuals to progressive disease [24]. Even so, it is currently unknown what effect FEH lesions coinfected with high-risk genotypes, such as HPV16 or HPV18, have on a possible malignant transformation, and the correlation to other HPV-positive oral indications, since they have not been systematically reported. This type of study is currently ongoing among indigenous Australians [25].

When multiple genotypes are at play, superinfection exclusion may limit or block secondary coinfections. Superinfection exclusion is an event in which a single cell infected by a specific type of virus is unable to become infected with a secondary virus of the same type. Hence, coinfection with more than one HPV genotype can be rare [26]. Although reports have been published showing that FEH lesions can be coinfected with multiple HPV genotypes [18], some level of superinfection exclusion might occur involving high-risk genotypes, such as HPV16 and HPV18. An in vitro study utilizing cervical keratinocyte cell lines showed that a pre-existing infection with the high-risk HPV16 was able to block or exclude a secondary high-risk HPV18 infection on the cell surface during the viral attachment phase. This resulted in a marked decrease in HPV18 infectivity after HPV16 had already infected the cell [26]. Still, cases of coinfection with HPV16 and HPV18 in cervical cancer cells on a single cell level were described [27], but an existing HPV infection might confer some protection against secondary infections with closely related genotypes.

## 3. Transmission and Diagnosis

The household transmission of FEH is believed to be through saliva, as free HPV13 viruses were detected in the saliva of infected individuals [28]. In a survey connected to this study, more than 70% of the study group, living in a rural community in the Mayan area of Mexico (*n* = 53, age 4–73 years, mean age 13.2 years), answered that they had experienced sharing toothbrushes and kitchen utensils, such as cups, cutlery and dishes, and only had access to poor sanitary conditions [28].

A diagnosis of FEH is often based solely on clinical presentation and histopathology, while the HPV involvement is rarely assessed [2,5,6,9,13,17,29]. Distinct pathognomonic findings include koilocytosis with clear cytoplasm, epithelial hyperplasia with parakeratosis and acanthosis, widened and thickened rete ridges, ballooning degeneration and mitosoid bodies (example of the latter in Figure 1c) [7,30]. Mitosoid bodies were frequently reported by Ledesma-Montes et al., who investigated 52 Mexican Mestizo patients (age 4–69 years old). However, the authors stated that the presence of mitosoid bodies in the biopsy material was not necessary to diagnose FEH [31].

In some instances, a pan-anti-HPV antibody is used for immunohistochemistry (IHC) on biopsy material. However, this gives no information regarding the specific genotype involved [8,11]. The practice includes the risk of differential diagnoses, such as verruca vulgaris or squamous cell papilloma, which are also known for their HPV involvement [32]. Furthermore, the use of standard kits for HPV genotyping often does not include HPV13 and HPV32 probes, maybe due to their low-risk and clinical asymptomatic manifestation. Previously, infection with HPV13 and HPV32 was determined via PCR analysis by utilizing specific primers [12,15,16] and, in some instances, followed by DNA sequencing [10,23,33]. However, biopsy material is not essential to determine an HPV infection; Conde-Ferráez, Borborema-Santos and colleagues demonstrated that enough biological material could be obtained using the cytobrush technique to collect oral exfoliate cells. This technique is non-invasive and therefore easier to perform on children and while in the field [10,23].

## 4. Geographical and Ethnical Distribution

FEH was primarily reported in the indigenous populations around the world, but the number of reported cases has been increasing in the European region. From 1966–2005, Sethi and colleagues reported nine published cases. In 2001–2019, this number had risen to 20 published cases [1]. From 2020–2021, two cases were reported within the European region [4,17], while several reports were published around the world, with many of them from the United States [8,9,11,15,34]. This increase in the registration of FEH cases is likely influenced by the individual healthcare systems in the varying countries; however, as the disease is mostly asymptomatic, cases cannot be expected to be systematically documented. The general health awareness and awareness about FEH, as well as an increase in immigration from countries with a higher number of reported cases, could also result in an increased registration within the European region.

In Greenland, the Danish medical doctor Helms described symptoms associated with FEH among the indigenous population back in 1894 [7], and lesions have since been reported in patients of, e.g., Sudanese [5], Turkish [4], Guatemalan [15], African-American [8], Saudi-Arabian [9], Mexican [2,6,18], Central Amazonian Indian [10], American Caucasian [11,14] and Argentinian [35] origin. In some ethnic groups, the prevalence is quite low, from 0.11% of the general Caucasian population in Sweden [36] and 0.09% for the province of Jujuy in Argentina (*n* = 2147) [35]; in contrast, it was reported to be 19.4% (*n* = 460, 46% of the inhabitants) among the indigenous Greenlanders from Nanortalik in southwest Greenland [37] and 32.3% in children of the Mexican Nahuatl ethnic group (*n* = 343) [2].

In many cases, FEH was described to affect several close relatives [2,5,6,10,18,20], even though the opposite was described too [4,10,11,12]. FEH was also associated with a low socioeconomic status [2,6,16,18,19,33]; families living close together [5] or living in a nonmetropolitan location [33]; sharing of food and personal objects, such as toothbrushes and utensils; and poor hygiene conditions [10,20,28]. It is important to note that many of these reports are based on clinical and/or histological examinations and did not always include HPV investigation. Finally, a specific allele of the human leukocyte antigen (HLA), HLA-DR4, was connected to an increased frequency of FEH in a smaller study cohort of 22 patients. HLA-DR4 frequently occurs among the indigenous populations of South America [19].

## 5. Comorbidities

There are several reports describing FEH as a comorbidity with other diseases. In particular, immunosuppressed patients seem to have a higher incidence compared to the general population. Diseases with a lower level of naïve CD4+ T-cells, such as HIV and intestinal lymphangiectasis, were described in relation to FEH [4,13,16,32]. In one of the HIV cases, the patient reported a remarkedly worsening of his oral condition as the lesions grew in both size and number following successful treatment of his HIV condition [16]. In a larger study of HIV-positive patients, 62% of the investigated patients (*n* = 29) had FEH, 26% of which were infected with HPV13 and 31% with HPV32 [32]. Other diseases that were reported with the presence of FEH include immunodeficient patients diagnosed with lymphopenia and hypogammaglobulinemia [15] and leukocyte adhesion deficiency [38], lung transplant [8], chronic graft versus host disease [34] and rheumatoid arthritis [14].

## 6. Prevention and Treatment

Preclinical research into FEH is complicated by the fact that the HPV life cycle is heavily dependent on host cell differentiation. Therefore, native HPV is only produced in vivo, while in vitro studies utilizing, e.g., immortalized cell lines rely on synthetic virus particles that can bypass the need for cell differentiation [39]. To this end, no preclinical models for HPV13 and HPV32 exist, but Ocadiz-Delgado et al. developed a transgenic murine model expressing the HPV16 E6/E7 oncogenes. At the age of 27 weeks, the mice spontaneously developed FEH in the middle tongue area [40], currently making this model the best preclinical option for studying FEH.

When it comes to clinical manifestations of FEH, in some instances, the characteristic nodules spontaneously disappear, leaving no residual functional or esthetic defects [5,6]. The duration of lesions was reported to last several weeks to more than 30 years [6,19], and many cases were reported to be asymptomatic, warranting no further treatment [2,5,6,8,9,10,11,12,13,14]. However, in order to prevent coinfection with high-risk HPV genotypes, a prophylactic HPV vaccination, which also includes some cross-protection against related genotypes, can be utilized [41]. Currently, three prophylactic vaccines are available that target the following low-risk and high-risk genotypes: a bivalent HPV16 and HPV18 vaccine (Cervarix); a four-valent HPV6, 11, 16 and 18 vaccine (Gardasil); and a nine-valent vaccine covering HPV6, 11, 16, 18, 31, 33, 45, 52 and 59 (Gardasil 9) [3]. The Gardasil vaccine was shown to induce both a humoral immune response with neutralizing antibodies residing in serum and oral fluids of the vaccinees and a cellular immune response consisting of cytotoxic CD8+ T cells [42,43]. As FEH is most prevalent in children and young adults [1], it is of utmost importance to vaccinate early in order to avoid these high-risk HPV coinfections. In many European countries, HPV vaccination was already implemented in the vaccination programs [44].

That the implementation of these vaccines has been vastly successful was shown by e.g., a Swedish study investigating the HPV vaccination effects, who found that the oral HPV prevalence decreased from ~10% in unvaccinated youth from 2009–2011 to <2% after 2013, where more than 70% of the individuals were vaccinated [45]. The prevalence of oral infections involving the genotypes covered by the HPV vaccines was significantly reduced in vaccinated versus unvaccinated young adults [46,47], while non-vaccine high-risk genotypes had a similar prevalence in the two study groups [47]. However, none of the approved vaccines currently include the most prevalent genotypes found in FEH, namely, HPV13 and HPV32 [3], even though some vaccine cross-protection between HPV strains with genomic similarity can be expected [41]. Thus, the impact of HPV vaccines on these low-risk genotypes is currently unknown.

In cases where the lesions grow to a size or at a location where treatment is warranted [15,16], treatment modalities include imiquimoid 5% cream [4,13,34], local cryotherapy using liquid nitrogen application [6], cauterization [10], surgical resection [29], ablative laser treatment [7,12,15,34], laser excision [48] and 80% trichloroacetic acid [49]. In a single report describing recurrence, imiquimod treatment was restarted, and the lesions consequently disappeared [4].

## 7. Conclusions

FEH is primarily associated with HPV13 and HPV32 infection, and coinfection with high-risk HPV16 and HPV18 was reported. HPV16 and HPV18 are covered by current HPV vaccines. However, the impact of the vaccines on infection with HPV13 and 32, and thus the overall prevalence of FEH, is currently unknown, but vaccinees might experience some cross-protection to related genotypes. Presently, the vaccines do not include HPV13 and HPV32.

For the first time, we present a histological picture of FEH with proven HPV90 involvement. The diagnosis of FEH is often based solely on clinical examination and histopathology, risking a differential diagnosis. HPV assessment is therefore recommended. Despite being described primarily among the indigenous populations around the world, a recent report has found the number of reported cases in the European region to be increasing. Hence, more research in the field is of utmost importance to establish the exact pattern of the spread of this disease.

## Data Availability

Not applicable.
